# 

**Published:** 1921-01-29

**Authors:** 


### Matrons' and Sisters' Appointments.

Bangor Borough Hospital.—Miss M. J. Winter has been appointed sister. She was trained at Bangor and at the Walkergate Hospital, Newcastle-on-Tyne, where she was the Gold Medallist of her year.

Hospital for Tuberculous Children, Graymount, Belfast.—Miss Letitia Barton has been appointed sister. She was trained at Belfast Union Infirmary, and was later staff nurse in the Union's fever hospital. She has since held the post of senior staff nurse at the Forster Green Hospital, Belfast, and sister at the Ophthalmic Hospital, Belfast.

Willesden General Hospital.—Miss Emily Brooker has been appointed matron. She was trained at the Norfolk and Norwich Hospital, and has been assistant matron of the West London Hospital.

Royal Albert Edward Infirmary and Dispensary, Wigan.—Miss S. Lunn has been appointed assistant matron. She was trained at the General Hospital, Nottingham, where she has since been sister and night superintendent, and where she also received special training in the house-keeping department.

### Forthcoming Events.

THE following Hunterian Lectures will be delivered in the theatre of the Royal College of Surgeons of England, at 5 P.M., upon the dates noted below:

MONDAY, JANUARY 31.—"The Pathology and Surgery of Lupus," by W. Sampson Handley, M.S., M.D., F.R.C.S.

WEDNESDAY, FEBRUARY 2.—"Mucocele and Pyocele of the Nasal Accessory Sinuses," by W. G. Howarth, M.A., M.B., F.R.C.S.

FRIDAY, FEBRUARY 4.—"Injuries of the Diaphragm, with special reference to wounds jointly involving thoracic and abdominal viscera," by C. W. Gordon Bryan, M.C., F.R.C.S.

